# Arbuscular mycorrhizal fungi improve the growth and drought tolerance of *Cinnamomum migao* by enhancing physio‐biochemical responses

**DOI:** 10.1002/ece3.9091

**Published:** 2022-07-11

**Authors:** Qiuxiao Yan, Xiangying Li, Xuefeng Xiao, Jingzhong Chen, Jiming Liu, Changhu Lin, Ruiting Guan, Daoping Wang

**Affiliations:** ^1^ Department of Ecology, College of Forestry Guizhou University Guiyang China; ^2^ The Key Laboratory of Chemistry for Natural Products of Guizhou Province and Chinese Academy of Sciences Guiyang China; ^3^ State Key Laboratory of Functions and Applications of Medicinal Plants Guizhou Medical University Guiyang China; ^4^ Institute of New Rural Development Guizhou University Guiyang China; ^5^ Department of Labor Health and Environmental Hygiene, School of Public Health Guizhou Medical University Guiyang China

**Keywords:** antioxidant system, arbuscular mycorrhizal fungi, *Cinnamomum migao*, drought stress, osmotic adjustment, photosynthesis, plant growth

## Abstract

Drought is the main limiting factor for plant growth in karst areas with a fragile ecological environment. *Cinnamomum migao* H.W. Li is an endemic medicinal woody plant present in the karst areas of southwestern China, and it is endangered due to poor drought tolerance. Arbuscular mycorrhizal fungi (AMF) are known to enhance the drought tolerance of plants. However, few studies have examined the contribution of AMF in improving the drought tolerance of *C. migao* seedlings. Therefore, we conducted a series of experiments to determine whether a single inoculation and coinoculation of AMF (*Claroideoglomus lamellosum* and *Claroideoglomus etunicatum*) enhanced the drought tolerance of *C. migao*. Furthermore, we compared the effects of single inoculation and coinoculation with different inoculum sizes (20, 40, 60, and 100 g; four replicates per treatment) on mycorrhizal colonization rate, plant growth, photosynthetic parameters, antioxidant enzyme activity, and malondialdehyde (MDA) and osmoregulatory substance contents. The results showed that compared with nonmycorrhizal plants, AMF colonization significantly improved plant growing status; net photosynthetic rate; superoxide dismutase, catalase, and peroxidase activities; and soluble sugar, soluble protein, and proline contents. Furthermore, AMF colonization increased relative water content and reduced MDA content in cells. These combined cumulative effects of AMF symbiosis ultimately enhanced the drought tolerance of seedlings and were closely related to the inoculum size. With an increase in inoculum size, the growth rate and drought tolerance of plants first increased and then decreased. The damage caused by drought stress could be reduced by inoculating 40–60 g of AMF, and the effect of coinoculation was significantly better than that of single inoculation at 60 g of AMF, while the effect was opposite at 40 g of AMF. Additionally, the interaction between AMF and inoculum sizes had a significant effect on drought tolerance. In conclusion, the inoculation of the AMF (*Cl. lamellosum* and *Cl. etunicatum*) improved photosynthesis, activated antioxidant enzymes, regulated cell osmotic state, and enhanced the drought tolerance of *C. migao*, enabling its growth in fragile ecological environments.

## Graphical abstract

The presence of AM fungi, *Claroideoglomus etunicatum* and *Claroideoglomus lamellosum*, improved the drought tolerance of *Cinnamomum migao* by increasing photosynthetic parameters, antioxidant enzyme activities, and osmosis‐regulating substances. In addition,the interaction between AMF and inoculum sizes had an obvious effect on drought tolerance. The results of this study supported the drought‐resistant mechanism of *C. migao* mediated by AMF.

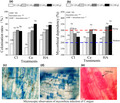

## 1 INTRODUCTION

Extreme weather, which is caused by global climate change, seriously threatens the survival of several living species on the whole Earth. Drought, one of the most serious natural disasters, has become an important concern for governments and scientists over the world (Wang et al., 2021). It greatly affects plant growth and distribution, and the degree of damage it causes is closely related to its severity and duration (Liu et al., 2011). However, the severity and duration of drought are unpredictable due to many reasons, such as precipitation occurrence and distribution, evaporation, and soil water storage capacity (Farooq et al., 2009). Therefore, drought has severe impacts on forest ecosystems, particularly fragile ecological environments like karst rocky desertification areas.

Karst lands, which are fragile ecological environments, are characterized by slow soil formation, shallow and discontinuous soil, low water holding capacity, low vegetation coverage, high soil erosion, and even serious rock desertification (Wang, Zhang et al., 2019; Zhang et al., 2021). They account for approximately 10% of the world's land surface (Hartmann et al., 2014). Additionally, the precipitation in karst areas is unevenly distributed in time and space (Cheng et al., 2018), causing regional and seasonal droughts (Guan et al., 2022). Altogether, the mechanism of drought in karst regions is complex and unpredictable (Cheng et al., 2018), which makes it difficult to study drought in such regions (Huang et al., 2008; Jiang et al., 2009). However, recently, under the background of global warming, the degree of drought in karst areas has shown an increasing trend, making drought stress an important factor limiting the survival and growth of plants (Guo, 2012).

Furthermore, drought stress adversely affects the physiology, biochemistry, growth, and development of plants worldwide. It leads to the accumulation of reactive oxygen species (ROS) in plants, destroys cell membranes, and disrupts the dynamic balance of active oxygen content (Komivi et al., 2017). These physiological and biochemical responses of plants under drought stress cause growth inhibition and even death (Fathi & Tari, 2016; Khan et al., 2020). Many studies have shown that the inoculation of mycorrhizal fungi initiates morphological, nutritional, and physiological changes in host plants to counter biotic and abiotic stresses and enhance plant growth and vigor (Ahmad et al., 2018; Brundrett & Tedersoo, 2018; Orians et al., 2017; Rajtor & Piotrowska‐Seget, 2016; Tamayo‐Velez & Osorio, 2017; Tran et al., 2019). Approximately 72% of the known vascular plants can act as hosts for arbuscular mycorrhizal fungi (AMF), and such mutually beneficial mycorrhizal associations have key roles in maintaining plant productivity in natural and agricultural habitats (Brundrett & Tedersoo, 2018; Huang et al., 2020; Smith & Read, 2008). AMF affect the vegetative roots of host plants that have not yet been lignified, penetrate and colonize the root to form highly differentiated symbiotic structures known as arbuscules, and form epitaxial mycelia and other fungal structures (Brundrett & Tedersoo, 2018; Genre et al., 2017; Harrison, 2005; Liu et al., 2006). This increases the root surface area (RSA), thereby improving the absorption range and capacity of water and nutrient elements (Guo et al., 2019; Li et al., 2018). Additionally, AMF can activate the defense mechanisms of plants against oxidative damage, induce increased antioxidant enzyme activity, clear harmful substances such as ROS and malondialdehyde (MDA) (Hou et al., 2018; Li et al., 2018), improve chlorophyll content, and promote photosynthesis (Sohrabi et al., 2012). Furthermore, AMF can adjust osmoregulatory substances and molecular signals and alter water metabolism (Golparyan et al., 2018; Lehto & Zwiazek, 2011; Sebastiana et al., 2018). These changes directly or indirectly enhance plant drought tolerance. Considering the characteristics of drought and nutrient deficiency in degraded karst ecosystem (Liu et al., 2008), inoculating AMF enhances plant growth by improving drought tolerance (Sebastiana et al., 2018), gradually restoring the function of the fragile karst ecosystem (Jiang et al., 2009). Notably, although AMF are beneficial to the growth of symbiotic plants (Sanders, 2003), there is a certain limit to the inoculum size. Because AMF promote nutrient absorption and growth in host plants, they also need to obtain carbohydrates from host plants (Brundrett & Tedersoo, 2018). Thus, excessive AMF inoculation can make host plants lose a substantial amount of nutrients, thereby affecting plant growth. On the contrary, inoculum size below the optimal level does not induce good mycorrhizal infection (Li et al., 2019). Generally, the optimal inoculum size is determined by the mycorrhizal colonization rate, plant growth, photosynthesis, and other physiological and biochemical indicators (Geng et al., 2016; Li et al., 2019; Wang, 2008; Xiong et al., 2009).

**FIGURE 1:**
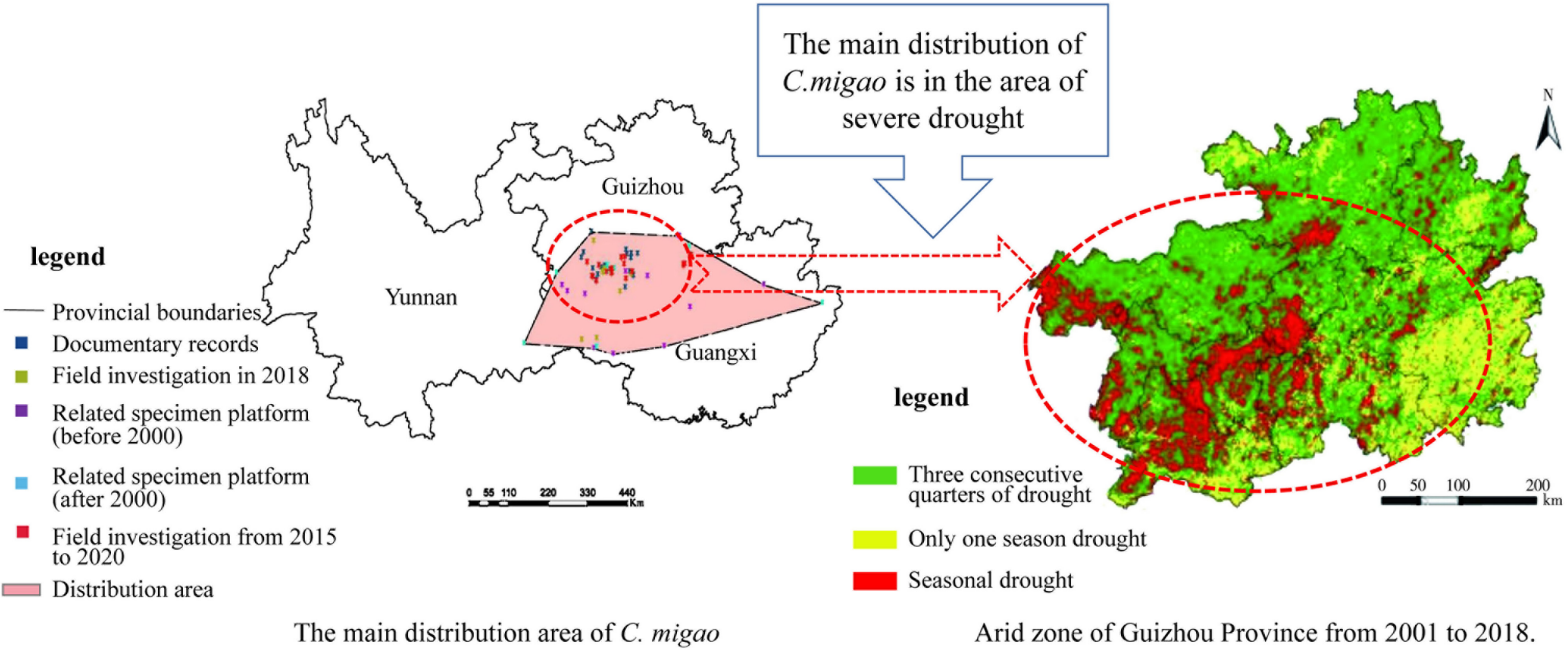
Main distribution area of *Cinnamomum migao* and arid zone of Guizhou Province during 2001–2018

*Cinnamomum migao* H. W. Li, a species of the Lauraceae family, is one of the endemic evergreen woody plants in the karst areas of southwestern China. The bark and wood of *C. migao* can be used as raw materials for making paper and wood slab. The fruit of *C. migao* contains a large amount of volatile oil and monoterpene or sesquiterpene chemicals; it is a traditional medicinal material of the Miao people in China and is used to cure gastrointestinal and cerebrovascular diseases (Huang et al., 2019; Liao et al., 2021). However, researchers discovered that the allelopathic and autotoxic nature of *C. migao* kept its population size small and range of species distribution narrow. Moreover, the low seed viability and germination rate, weak seedling formation, slow natural reproduction rate, and poor drought tolerance resulted in a low dispersal ability (Chen, 2020; Figure 1). Many natural populations of *C. migao* have disappeared, greatly threatening the survival and reproduction of this species (Huang et al., 2019). Additionally, seasonal and geological droughts in the main distribution areas lead to poor growth in the seedling stage (Figure 1), aggravating the survival crisis of *C. migao* in fragile karst areas (Chen, 2020; Cheng et al., 2018; Dai & Zhong, 2021).

To improve the drought tolerance of plants in karst forest ecosystems with frequent droughts, scientists have conducted substantial research on the mechanism of interaction between AMF and plants (Lehnert et al., 2018; Sanders, 2003; Sohrabi et al., 2012). However, to date, the influence of AMF on the growth and stress resistance of *C. migao* has not been studied, and the symbiotic effect is not clear. We propose the hypotheses that the inoculation of AMF strains promotes the growth and drought tolerance of *C. migao*. Therefore, in this study, two strains of *Glomus* were inoculated individually and collectively to analyze any changes in the drought tolerance of *C. migao* in karst soil (calcareous soil). The effects of inoculating AMF, *Claroideoglomus etunicatum* and *Claroideoglomus lamellosum*, on *C. migao* seedlings in four inoculum sizes (20, 40, 60, and 100 g) were also evaluated. In particular, we studied the effect of inoculation on the physiological processes in *C. migao* by analyzing several physiological indices, such as colonization rate (CR); biomass (DW); growth indices; photosynthetic indices; chlorophyll relative content value (SPAD); relative water content (RWC); total soluble sugar (TSS), soluble protein (SP), proline (Pro), and MDA contents; and catalase (CAT), peroxidase (POD), and superoxide dismutase (SOD) activities. We believe that this study can help elucidate the mechanism underlying AMF‐induced drought tolerance in *C. migao* and provide a scientific basis for AMF inoculation for artificial vegetation restoration in karst mountains.

## 2 MATERIALS AND METHODS

### 2.1 Experimental site

The pot experiment was conducted in the greenhouse of the College of Forestry of Guizhou University, Guiyang, Guizhou Province, China (106°420 E, 26°340 N, 1020 M A.S.L.). This area lies in the subtropical monsoon climate zone with an annual average temperature of 15.3°C and an annual average precipitation of 1129 mm.

### 2.2 Experimental materials

#### 2.2.1 Preparation of the inoculum

Arbuscular mycorrhizal fungi, *Cl. lamellosum* and *Cl. etunicatum*, were provided by the Bank of Glomeromycota in China (http://www.agridata.ac.cn/Web). *Trifolium repens* was used for cultivation and propagation. The cultivation substrate consisted of sand (0.7–1.0 mm in diameter) and perlite (2–4 mm in diameter) mixed at a ratio of 5:1, which was sterilized by autoclaving (30 min at 121°C, 103.4 kPa steam pressure) to eliminate all microorganisms. The substrate was used to fill two third of a sterilized plastic pot (wiped with 75% alcohol), after which a thin layer of microbial inocula (*Cl. lamellosum* and *Cl. etunicatum*) was added. Sterilized *T. repens* seeds (soaked in 10% H_2_O_2_ for 10 min and rinsed four times with sterile water) were then planted and overlaid with a 2‐cm thick layer of the sterilized substrate. The entire pot was then transferred to an artificial climate chamber (RXZ‐1500; Ningbo Jiangnan Instrument Factory, Ningbo, China) with controlled conditions of 25°C/20°C day/night temperature, >60% relative humidity, and a photoperiod of 14 h of artificial light (8000–10,000 Lux) and 10 h of darkness. The seeds were watered once per week with 60 ml of sterile Hoagland's nutrient solution (low phosphorus). After 128 days of culture, the roots of the *T. repens* seedlings were densely packed in the entire container, and the pot was dried for 1 week in a shady and ventilated room with stable temperature (20–25°C) and humidity (55–60%) after the shoots of the *T. repens* seedlings were removed. A 1‐cm layer of substrate was removed, and all the cultures (including *T. repens* roots, hyphae, spores, and substrates) were poured onto clean copier papers on a clean table top (all wiped with 70% alcohol). The plant parts were subsequently chopped with a flame‐sterilized hatchet. Finally, the chopped cultures were rolled up in paper and poured into sterile ziplock bags (wiped with 70% alcohol), which were sealed and stored in a refrigerator (4°C). These were used as fresh mycorrhizal inocula.

Sterile gloves were worn throughout the processes of cultivation and propagation to ensure that the cultures were not handled with bare hands. The inocula of *Cl. lamellosum* contained 19 spores per gram, whereas those of *Cl. etunicatum* contained 21 spores per gram, as the spores were isolated by wet screening combined with sucrose centrifugation and determined by stereomicroscopy (Liu & Li, 2000).

#### 2.2.2 Nursery substrate material

The seedling culture medium was mixed with forest soil (screened using a 10‐mesh sieve), river sand (with particle size of 0.7–1 mm), and perlite (with particle size of 2–4 mm) at a volume ratio of 5:1:1. Subsequently, it was sterilized continuously for 2 h under 0.14 MPa pressure and 125°C temperature. The basic physical and chemical properties of the soil were as follows: pH 6.6, soil organic matter 220.9 g/kg, total phosphorus 2.6 g/kg, total nitrogen 7.96 g/kg, total potassium 18.4 g/kg, alkali‐hydrolyzable nitrogen 31.4 mg/kg, available phosphorus (extracted with 0.5 mol/L NaHCO_3_) 1.6 mg/kg, and available potassium (extracted with 1 mol/L NH_4_OAc) 568.0 mg/kg. The culture container was soaked in 75% ethanol for 30 min and then rinsed with sterile water for later use.

#### 2.2.3 Plant materials

From October to November 2018, fresh fruits of *C. migao* were collected from Luodian County, Guizhou Province (25°260 N, 106°310 E, 761 M A.S.L.). Subsequently, they were transported to the laboratory, and their flesh was immediately removed and washed with water. Before germination, the seeds were cleaned, disinfected by soaking in 0.5% KMnO_4_ for 2 h, and then washed five times with sterile water. The seeds were treated with 200 mg/L gibberellin solution for 48 h, sterilized by stirring with 5% sodium hypochlorite for 10 min, washed four times with sterile water, planted in sterilized river sand, and then transferred to an artificial climate chamber (RXZ‐1500) with a germination box under the controlled conditions of 25°C/20°C day/night temperature, >40% relative humidity, and a 12/12‐h light cycle comprising 12 h of light (8000–10,000 Lux) and 12 h of darkness; they were regularly irrigated with deionized water. After the seeds germinated, the seedlings were moved out of the artificial climate chamber and grown for 30 days in a greenhouse.

### 2.3 Experimental design

The experimental design is shown in Table 1. It included the following three AMF inoculation treatments: *Cl. etunicatum* (Ce), *Cl. lamellosum* (Cl), and *Cl. etunicatum + Cl. lamellosum* (HA). None of the inoculation treatments (NM) were conducted with the same quantity of inoculum containing AMF‐free filtrate. Four inoculum sizes – 20 g (S1), 40 g (S2), 60 g (S3), and 100 g (S4) – were maintained for each AMF inoculation treatment, for a total of 16 treatments, and four replicates (four pots) per inoculum treatment (*n* = 4).

**TABLE 1:** Experimental design

Treatments	Detailed instructions	Inoculum sizes (g)			
		S1	S2	S3	S4
NM	*Claroideoglomus etunicatum* and *Claroideoglomus lamellosum* inactivated by high temperature	20	40	60	100
Ce	*Claroideoglomus etunicatum*	20	40	60	100
Cl	*Claroideoglomus lamellosum*	20	40	60	100
HA	*Claroideoglomus etunicatum + Claroideoglomus lamellosum*	20	40	60	100

### 2.4 Greenhouse study

The pot‐culture experiment was started in March 2019. The seedlings were grown under natural light in a plastic greenhouse (annual average temperature: 18.7°C, annual mean humidity: 60%, and annual average illumination: 1148.3 h) after the seedlings had been transplanted into sterilized plastic plots. *Cinnamomum migao* seedlings with relatively consistent growth were selected, rinsed with detergent, immersed in 1% NaClO solution for 2–3 min, and rinsed with sterile water. First, 2.5 kg of sterilized soil was added to the pot and flattened, following which the microbial inocula were evenly added. Subsequently, a sterilized seedling was added, followed by the addition of 1 kg of sterilized soil to complete the planting. For a total of 64 pots, each pot had one seedling.

After 120 days of growth under normal water supply conditions, *C. migao* seedlings were subjected to drought stress for 30 days and then the physiological and biochemical parameters were measured. Soil moisture content was measured by a soil moisture meter (ECA‐SW1) and maintained at 80–90% of the field capacity by weighing the pot and watering daily. Watering was discontinued after 120 days, and drought stress treatment was initiated for 30 days when the soil moisture content in the pots naturally reached 50%. The soil moisture content was measured and maintained at 50% daily.

### 2.5 Plant harvest and analysis of physiological and biochemical parameters

Before harvest, shoot height (Sh) and stem diameter (Sd) were measured using a ruler and vernier calipers, and leaf area (La) of the fourth and fifth leaves were measured using a La meter (LI‐3100, LI‐COR, USA), a total of eight leaves were measured for each treatment. The SPAD value of the relative content of chlorophyll was measured using a portable SPAD‐502 chlorophyll meter (Konica Minolta, Japan). The photosynthetic parameters (including net photosynthetic rate [Pn], transpiration rate [Tr], stomatal conductance [Gs], intercellular CO_2_ concentration [Ci]) of the fourth fully unfolded leaf counted from the top of *C. migao* plants were determined at room temperature (25°C) using a portable photosynthesis system (LI‐6800, LI‐COR). The measurements were performed at a light intensity of 1000 μmol (photon)/m^2^/s from 9:00 to 11:00 a.m. The CO_2_ concentration in the sample chamber was 400 μmol/mol with a flow rate of 500 μmol/s. The leaf temperature was 25 ± 0.8°C, and relative humidity was 60%. Water‐use efficiency (WUE) was calculated as the ratio between Pn and Tr.

Following this, the plants were harvested and divided into roots, stems, and leaves. Their fresh weights were subsequently measured using a scale. Roots of *C. migao* seedlings were subjected to a root image scanner (Epson7500, resolution: 600 dpi), and the root length (Rl), root surface area (RSA), and the number of connections (Ncon), tips (Ntip), furcation (Nf), and crossing (Ncro) of roots were analyzed using WinRHIZO Pro LA2400 root analysis system (Regent Instruments, QC, Canada). Some fresh samples were immediately put in liquid nitrogen and then stored in a low‐temperature refrigerator (−80°C) for further use, whereas the remaining were dried in an oven to measure dry weight. Biomass was calculated in terms of the dry weight of the whole plant.

The mycorrhizal root CR was determined according to the method described by Phillips and Hayman (1970). Four plants were determined for each treatment. Briefly, 1‐cm segments were cut from the middle part of the root (180 root segments for each treatment), washed with 10% (w/v) KOH at 90°C for 30 min, decolorized with 10% H_2_O_2_, and stained with 0.05% (w/v) trypan blue and 0.05% (w/v) acid fuchsin in lactophenol. Stained root tissues were examined under a light microscope (CX43, Olympus, Tokyo, Japan). The mycorrhizal root CR was expressed in terms of the length of unit root infection:

$$\mathrm{CR}=\frac{\mathrm{CRL}}{\mathrm{RL}}\times 100\%,$$

where CRL represents the length of the colonized root segment and RL represents the total root length.

Mycorrhizal dependency (MD) was determined by Menge et al.’s (1978) method:

$$\mathrm{MD}=\frac{\mathrm{MDW}}{\mathrm{NDW}\;}\times 100\%$$

where MDW represents the total biomass of AM plants and NDW represents the total biomass of NM plant. In general, MD was divided into three levels – MD ≤100%, indicating weak or no dependence on mycorrhiza; MD ≥200%, indicating medium dependence on mycorrhiza; and MD ≥300%, indicating strong dependence on mycorrhiza.

The frozen plant leaves were taken out of liquid nitrogen, and the corresponding indices were determined according to the following methods: SOD activity was determined by measuring the amount of reduced nitroblue tetrazolium (Gao, 2006); CAT activity was determined by measuring the reduction in hydrogen peroxide absorbance at 240 nm using the ultraviolet absorption method (Becana et al., 1986; Britton & Machlly, 1956); POD activity was determined by tracking the alteration in POD activity at 470 nm for 5 min using the guaiacol method (Li et al., 2000); proline content was determined colorimetrically via the ninhydrin method previously described by Li et al. (2000); TSS content was determined colorimetrically via the anthrone method (Gao, 2006); SP content was determined using the Coomassie Brilliant Blue G‐250 method (Bradford, 1976); and MDA content was determined using the thiobarbituric acid method (Gao, 2006). Relative water content (RWC) was determined using the following formula (Gao, 2006):

$$\mathrm{RWC}=\frac{\mathrm{FW}-\mathrm{DW}}{\mathrm{SFW}-\mathrm{DW}}\times 100\%,$$

where FW, DW, and SFW represent fresh weight, dry weight, and saturated fresh weight, respectively. SFW was measured after the leaves were completely immersed in water for 24 h in darkness at 4°C.

### 2.6 Statistical analysis

Data were tested for normality (Shapiro–Wilk test, *p* > .05) and homogeneity of variances. Values are expressed as mean ± *SE* (plot replicates, *n* = 4). The data were subjected to analysis of variance (ANOVA) and factor analysis using SPSS 21.0 statistical software package (Chicago, IL, USA), and differences between the mean values were compared using the least significant differences (LSD) post hoc test. *p* Values of <.05 were considered statistically significant. Correlation coefficients between variables were tested using Pearson's correlation. Graphs were created using OriginPro 9.0 (Origin Lab in Northampton, MA, USA), Photoshop (Adobe, USA), and R language software (R version 4.1.2).

## 3 RESULTS

### 3.1 Mycorrhizal root CR and MD

**FIGURE 2:**
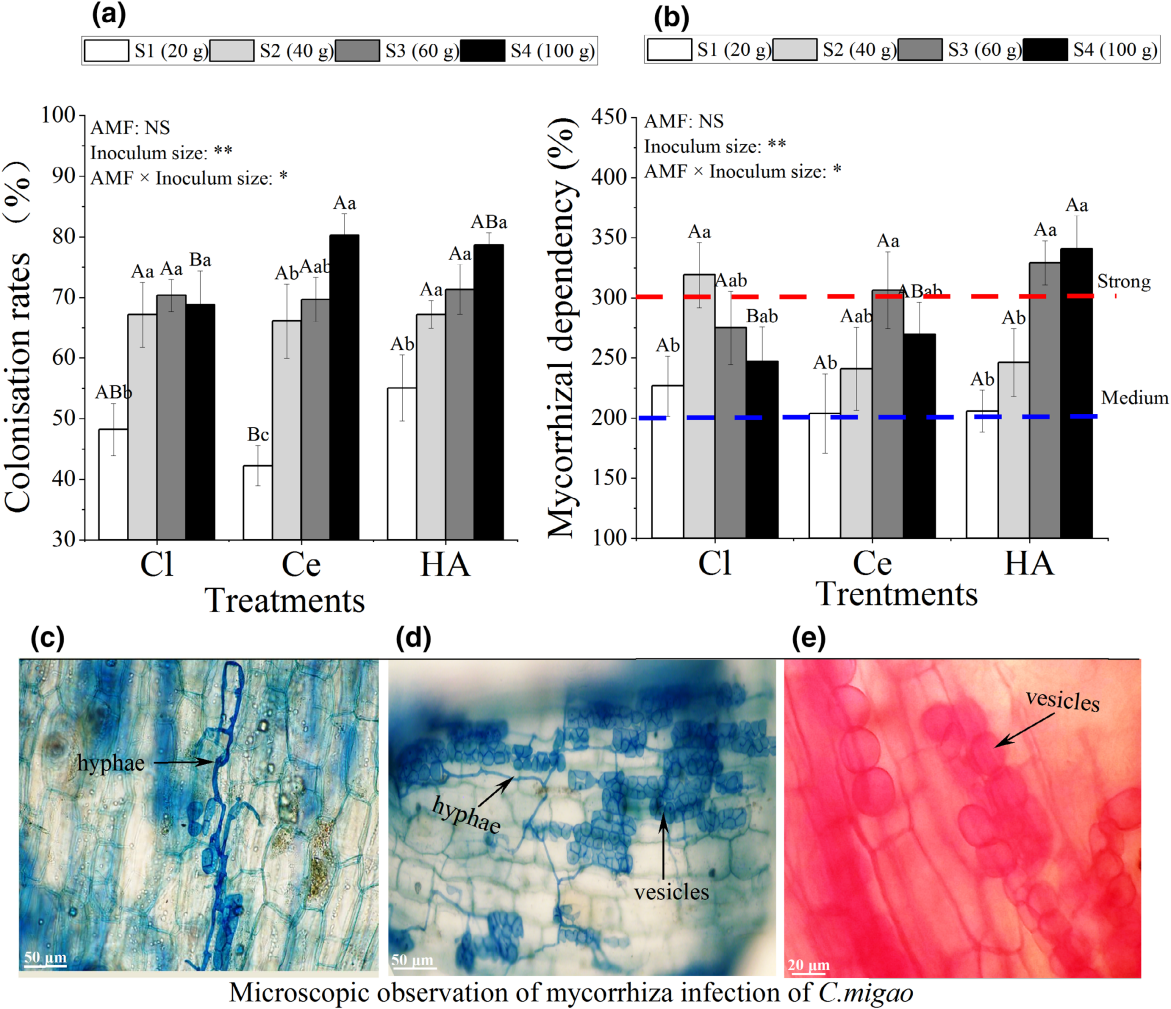
(a) The mycorrhizal root colonization rates. (b) The mycorrhizal dependency. Cortical tissue was infected, revealing (c) the presence of hyphae formed by the inoculated *Claroideoglomus etunicatum*; (d) hyphae and vesicles formed by the inoculated *Claroideoglomus lamellosum*; and (e) vesicles formed by the coinoculation of *Cl. lamellosum* and *Cl. etunicatum. Note*: Vertical bars represent the standard errors of the means based on four replicates. Different lowercase letters indicate significant differences between different inoculum sizes in the same AMF treatment, whereas different capital letters indicate significant differences between different AMF treatments with the same inoculum size (LSD test; *p* < .05). Significant differences at **p < .001 and * *p* < .05, and NS indicates no significant differences

Roots of all AM plants possessed intraradical fungal colonization (Figure 2a–e), whereas NM plants were free of fungal colonization. The results of a two‐factor ANOVA showed that the inoculation of different AMF (Cl, Ce, and HA) had no significant effect on CR and MD. The interaction between inoculum sizes and AMF inoculations had a significant effect on CR and MD (*p* < .05; Figure 2a,b). The CR in all treatments was significantly positively correlated with inoculum sizes (*p* < .001; Figure 2a). MD of HA treatment improved with an increase in inoculum size, but the MD of Cl and Ce treatments increased first and then decreased. The MD in all treatments was either medium or strong (Figure 2b).

### 3.2 Growth of *C. migao* seedlings

Compared with NM plants, the inoculation of AMF had a positive effect on the growth of *C. migao* seedlings. Compared with the biomass (DW) of NM plants, the biomass of Cl, Ce, and HA plants was greater by 125–206%, 103–189%, and 112–234%, respectively. Among the three AMF inoculation groups, the highest biomass was observed in Cl‐S2 (10.20 ± 1.53 g), Ce‐S3 (9.01 ± 1.44 g), and HA‐S3 (10.21 ± 0.73 g) (Table 2). The Sh, Sd, and La of all AM plants were greater than those of NM plants, except the Sh of the NM‐S4 was greater than the that of Cl‐S4 and Ce‐S4, and the La of NM‐S1 was greater than that of Cl‐S1 (Table 2). With an increase in the inoculum size, these parameters first increased and then decreased. For the underground parts, except the RSA of Cl decreased with increasing the inoculum size, Rl, RSA, and root dry weight (RDW) (Table 2) as well as the Ncon, Ntip, Ncro, and Nf of the roots (Table 3) showed the same trend, indicating that within appropriate inoculum size, the inoculation of AMF promoted root elongation and bifurcation to form root networks, which was beneficial in terms of retaining soil moisture and absorbing nutrients.

**TABLE 2:** Plant growth indices of nonmycorrhizal (NM) and inoculated (AMF) *Cinnamomum migao* seedlings

Treatments	Inoculum size (size)/(g)	Shoot height (Sh)/(cm)	Stem diameter (Sd)/(mm)	Leaf area (La)/(cm^2^)	Root length (Rl)/(cm)	Root surface area (RSA)/(cm^2^)	Root DW (RDW)/(g)	Total DW (DW)/(g)
NM	20	22.13 ± 3.01^Ba^	4.23 ± 0.08^Aa^	15.79 ± 1.19^Bb^	679.61 ± 35.69^Cb^	182.48 ± 33.60^Cb^	0.91 ± 0.08^Ba^	3.77 ± 0.037^Ca^
	40	24.21 ± 3.01^Ca^	4.01 ± 0.15^Ca^	15.79 ± 2.31^Cb^	884.91 ± 39.93^Db^	349.98 ± 85.61^Ba^	1.00 ± 0.09^Ba^	3.33 ± 0.75^Cab^
	60	23.02 ± 3.04^Ca^	4.34 ± 0.07^Ca^	14.27 ± 7.50^Cc^	575.65 ± 51.47^Bc^	153.54 ± 10.78^Cb^	1.02 ± 0.38^Ba^	3.11 ± 0.26^Cab^
	100	26.03 ± 3.01^Ba^	4.40 ± 0.11^Ba^	16.32 ± 2.10^Aa^	1083.21 ± 50.50^Aa^	226.94 ± 77.32^Bab^	1.08 ± 0.17^Ba^	2.92 ± 0.79^Cb^
Cl	20	30.30 ± 1.44^Ab^	4.66 ± 0.49^Ab^	15.61 ± 2.99^Bb^	1810.77 ± 184.18^Aa^	832.44 ± 70.45^Aa^	1.12 ± 0.12^Bb^	8.50 ± 0.58^Ab^
	40	36.36 ± 1.40^Aa^	5.52 ± 0.30^Aa^	31.95 ± 4.31^Ba^	2013.55 ± 250.29^Ba^	744.86 ± 80.07^Aa^	1.85 ± 0.05^Aa^	10.20 ± 1.53^Aa^
	60	25.75 ± 1.32^BCc^	5.46 ± 0.29^Aa^	38.98 ± 5.13^Ba^	1316.45 ± 66.62^Aa^	538.29 ± 64.22^Ab^	1.71 ± 0.62^Aa^	8.48 ± 0.56^Bb^
	100	22.38 ± 0.29^Bd^	4.44 ± 0.11^Bb^	18.14 ± 4.49^Ab^	1212.82 ± 177.44^Aab^	494.95 ± 25.39^Ab^	1.45 ± 0.18^Aa^	7.17 ± 0.61^Bb^
Ce	20	24.83 ± 1.83^Bb^	4.66 ± 0.42^Aa^	17.92 ± 7.22^Bb^	1194.55 ± 42.84^Bc^	656.77 ± 95.02^Ba^	1.53 ± 0.04^Aa^	7.66 ± 0.71^Bb^
	40	36.20 ± 1.17^Aa^	4.89 ± 0.30^Ba^	45.86 ± 14.03^Aa^	2314.53 ± 238.63^Aa^	837.15 ± 149.17^Aa^	1.38 ± 0.78^ABa^	8.10 ± 0.66^Bb^
	60	26.48 ± 0.80^Bb^	5.20 ± 0.48^ABa^	51.02 ± 14.17^Aa^	1503.21 ± 69.82^Ab^	626.86 ± 103.46^Aa^	2.13 ± 0.27^Aa^	9.01 ± 1.44^ABa^
	100	25.33 ± 3.90^Bb^	4.86 ± 0.40^Aa^	24.42 ± 5.71^Ab^	681.29 ± 78.53^Bd^	184.39 ± 48.38^BCb^	0.94 ± 0.23^Bb^	7.97 ± 0.84^Bb^
HA	20	29.08 ± 2.04^Ab^	4.64 ± 0.13^Aa^	32.56 ± 9.94^Aa^	822.67 ± 123.08^Cb^	192.90 ± 37.96^Cc^	1.05 ± 0.13^Bb^	8.02 ± 0.75^ABc^
	40	29.30 ± 1.52^Bb^	5.12 ± 0.15^Ba^	42.69 ± 5.35^ABa^	1595.17 ± 157.87^Ca^	729.39 ± 179.01^Aa^	1.71 ± 0.33^Aa^	7.86 ± 0.74^Bc^
	60	41.90 ± 1.72^Aa^	4.96 ± 0.17^Ba^	38.98 ± 4.43^Ba^	1473.19 ± 277.51^Aa^	397.76 ± 68.46^Bb^	1.02 ± 0.08^Bb^	10.21 ± 0.73^Aa^
	100	30.75 ± 0.26^Ab^	4.63 ± 0.16^ABa^	18.17 ± 0.73^Ab^	532.07 ± 35.57^Cb^	127.59 ± 21.64^Cc^	1.39 ± 0.09^Aab^	9.76 ± 0.79^Ab^

*Note*: Data are shown as mean ± *SE* of four replicates for each treatment. Different lowercase letters indicated after the means in the same column signify significant differences between different inoculum sizes in the same AMF treatment (*p* < .05) and different capital letters in the same column signify significant differences between different AMF treatments with the same inoculum size (*p <* .05) by LSD test.

**TABLE 3:** Root morphology indices of nonmycorrhizal (NM) and inoculated (AMF) *Cinnamomum migao* seedlings

Treatments	Inoculum size (size)/(g)	Connection number of root (Ncon)	Number of root tip (Ntip)	Number of root furcation (Nf)	Crossing number of root (Ncro)
NM	20	1308 ± 160^Db^	593 ± 175^Bb^	607 ± 38^Db^	50 ± 10^Cb^
	40	1149 ± 193^Bb^	363 ± 41^Bc^	613 ± 125^Bb^	23 ± 3^Bb^
	60	2092 ± 412^Ca^	691 ± 113^Bb^	1068 ± 197^Ba^	70 ± 25^Bab^
	100	2053 ± 275^Ba^	940 ± 80^Aa^	1064 ± 127^Ba^	82 ± 21^Ba^
Cl	20	3744 ± 388^Ab^	1290 ± 303^Aa^	1875 ± 211^Ab^	141 ± 38^Ab^
	40	9990 ± 1004^Aa^	1414 ± 577^Aa^	5559 ± 583^Aa^	460 ± 65^Aa^
	60	2333 ± 418^Cc^	550 ± 185^Bb^	1216 ± 240^Bc^	114 ± 35^Bb^
	100	1973 ± 161^Bc^	950 ± 246^Aab^	962 ± 36^Bc^	35 ± 7^Cc^
Ce	20	2118 ± 358^Cb^	675 ± 100^Bb^	1093 ± 172^Cb^	67 ± 22^Cb^
	40	9131 ± 2033^Aa^	1286 ± 357^Aa^	5622 ± 1045^Aa^	513 ± 107^Aa^
	60	3500 ± 776^Bb^	821 ± 790^Bb^	2006 ± 370^Bb^	127 ± 51^Bb^
	100	3307 ± 479^Ab^	1012 ± 246^Aab^	2003 ± 503^Ab^	162 ± 40^Ab^
HA	20	2877 ± 362^Bb^	780 ± 160^Bc^	1394 ± 119^Bb^	101 ± 17^ABb^
	40	2974 ± 336^Bb^	1188 ± 305^Aab^	1405 ± 324^Bb^	87 ± 31^Bb^
	60	6037 ± 593^Aa^	1446 ± 232^Aa^	4817 ± 1219^Aa^	442 ± 143^Aa^
	100	2038 ± 259^Bb^	826 ± 76^Abc^	990 ± 192^Bb^	58 ± 19^BCb^

*Note*: Data are shown as means ± *SE* of four replicates for each treatment. Means followed by different lowercase letters in the same column indicate significantly different between different inoculum sizes under the same AMF treatments (*p* < .05), and different capital letters in the same column indicate significant differences between different AMF treatments under the same inoculum size (*p* < .05) by LSD test.

### 3.3 Photosynthetic parameters

**FIGURE 3:**
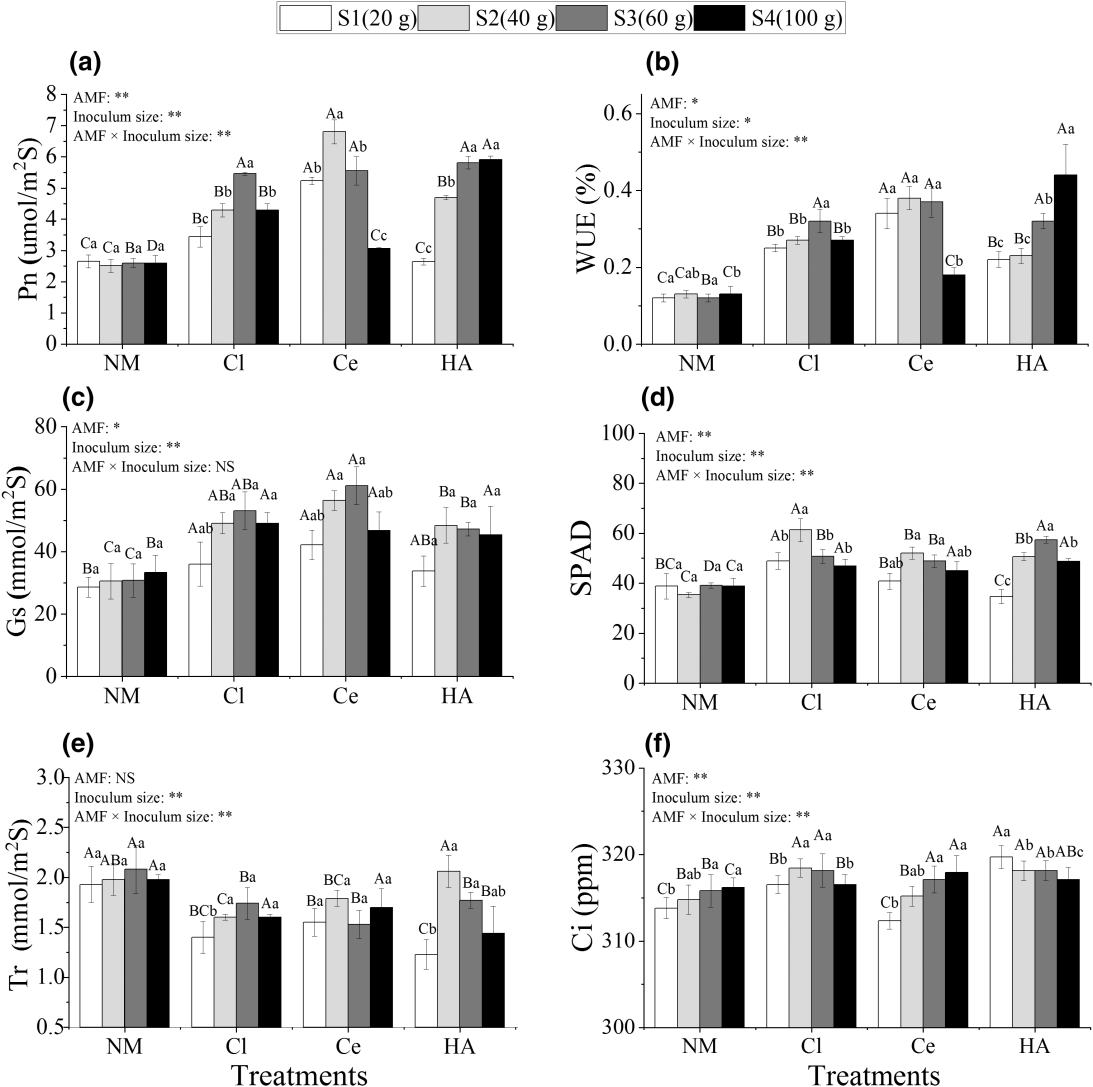
Net photosynthetic rate (Pn) (a), water use efficiency (WUE) (b), stomatal conductance (Gs) (c), SPAD (d), transpiration rate (Tr) (e), and intercellular CO_2_ concentration (Ci) (f) in *Cinnamomum migao* seedlings treated with different inoculation treatments (NM, Cl, Ce, and HA) and different inoculum sizes (S1, S2, S3, and S4). *Note*: Vertical bars represent the standard errors of the means based on four replicates. Different lowercase letters indicate significant differences between different inoculum sizes in the same AMF treatment, whereas different capital letters indicate significant differences between different AMF treatments with the same inoculum size (LSD test; *p* < .05). Significant differences at **p < .001 and *p < .05, and NS indicates no significant differences

Single or coinoculated *Cl. lamellosum* and *Cl. etunicatum* significantly affected the photosynthesis of *C. migao* seedlings. The net Pn (Figure 3a), WUE (Figure 3b), Gs (Figure 3c), SPAD (Figure 3d), and Ci (Figure 3f) of most AM plants were significantly higher than those of NM plants (*p* < .05), and Tr (Figure 3e) of NM plants was higher than that of AM plants. The Pn of HA plants increased with an increase in the inoculum size, whereas that of both Ce and Cl plants increased first and then decreased. The highest values were observed in Cl‐S3 (5.5 ± 0.1 μmol/m^2^/s), Ce‐S2 (6.8 ± 0.4 μmol/m^2^/s), and HA‐S4 (6.0 ± 0.1 μmol/m^2^/s) (Figure 3a). The WUE of Cl and Ce plants (Figure 3b), Gs (Figure 3c), SPAD (Figure 3d), and Tr (Figure 3e) of the three AM plants and Ci of Cl plants (Figure 3f) increased first and then decreased with an increase in the inoculum size, whereas the Ci of HA plants decreased (Figure 3f) and the WUE of HA plants increased (Figure 3b).

### 3.4 Antioxidant enzyme activity

**FIGURE 4:**
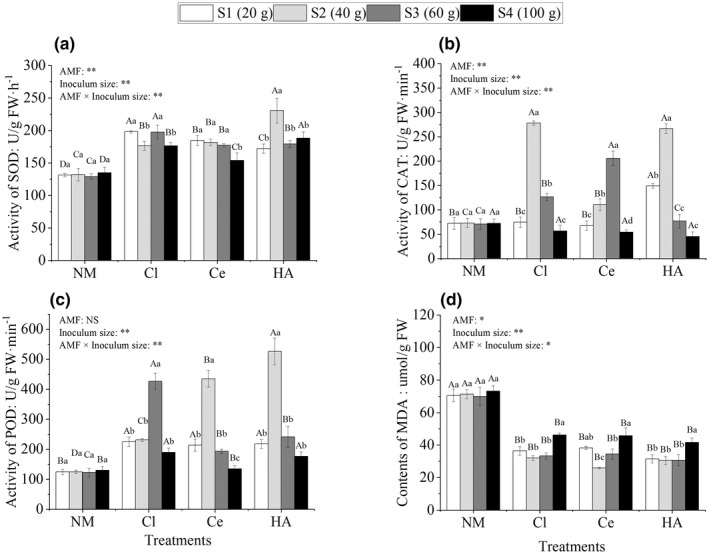
Superoxide dismutase (SOD) activity (a), catalase (CAT) activity (b), peroxidase (POD) activity (c), and malondialdehyde (MDA) content (d) in *Cinnamomum migao* seedlings treated with different inoculation treatments (NM, Cl, Ce, and HA) and different inoculum sizes (S1, S2, S3, and S4). *Note*: Vertical bars represent the standard errors of the means based on four replicates. Different lowercase letters indicate significant differences between different inoculum sizes in the same AMF treatment, whereas different capital letters indicate significant differences between different AMF treatments with the same inoculum size (LSD test; *p* < .05). Significant differences at **p < .001 and *p < .05, and NS indicates no significant differences

Superoxide dismutase activity in AM plants ranged from 153.9 ± 11.1 to 230.56 ± 11.9 U/g FW/h, which was significantly higher than that in NM plants by 13.9–74.5% (*p* < .05; Figure 4a). SOD activity in HA‐S2 plants (230.6 ± 11.9 U/g FW/h) was the highest among AM plants, whereas that in Ce‐S4 plants (153.9 ± 11.1 U/g FW/h) was the lowest (Figure 4a). CAT activity in AM plants ranged from 45.2 ± 11.2 to 278.0 ± 3.2 U/g FW/min (Figure 4b). With an increase in the inoculum size, CAT activity first increased and then decreased, with significant differences among four inoculum sizes. CAT activity in Cl‐S2, Cl‐S3, Ce‐S2, Ce‐S3, HA‐S1, and HA‐S2 plants was significantly higher than that in NM plants by 280.2%, 78.1%, 51.5%, 189.4.8%, 106.3%, and 264.8%, respectively (Figure 4b). Furthermore, CAT activity in Cl‐S4, Ce‐S4, and HA‐S4 plants decreased by 22.2%, 25.3%, and 37.7%, respectively (Figure 4b). As shown in Figure 4c, POD activity in AM plants ranged from 135.0 ± 7.1 to 526.7 ± 29.7 U/g FW/min. Except Ce‐S4, POD activity in the three AM plants was significantly higher than that in NM plants (*p* < .05; Figure 4c), and POD activity in Ce‐S2, HA‐S2, and Cl‐S3 plants increased by 356.5%, 321.3%, and 246.9%, respectively (Figure 4c).

### 3.5 MDA content

As shown in Figure 4d, MDA content in NM plants ranged from 69.9 ± 5.6 to 73.2 ± 3.3 μmol/g FW and that in AM plants ranged from 26.0 ± 0.3 to 46.2 ± 0.9 μmol/g FW. MDA content in AM plants was significantly lower than that in NM plants by 36.9–63.6%. Different AMF, inoculum sizes, and their interaction had significant effects on MDA content (*p* < .05 and *p* < .001).

### 3.6 RWC and TSS, SP, and Pro contents

**FIGURE 5:**
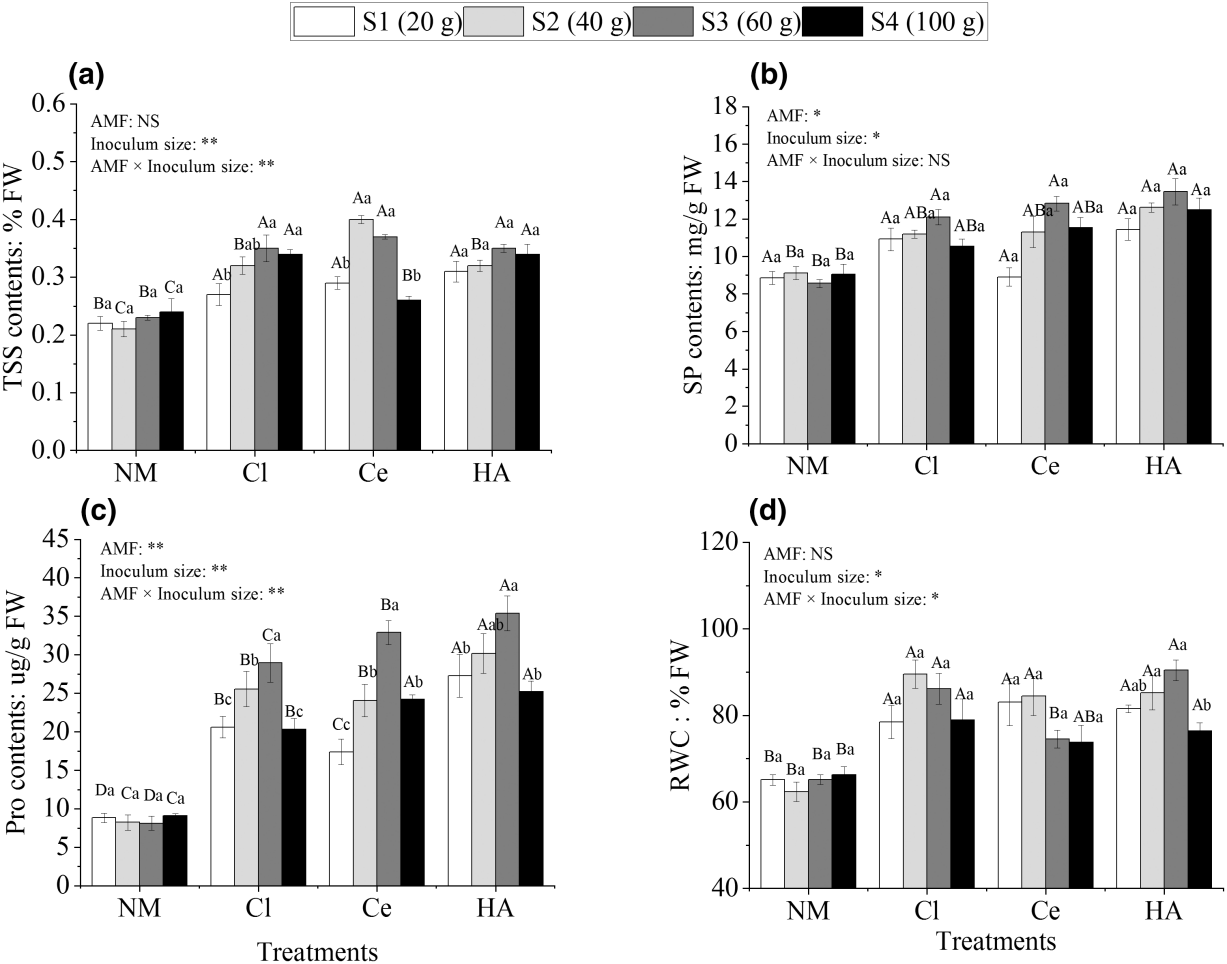
Total soluble sugar (TSS) (a), soluble protein (SP) (b), proline (Pro) (c), and relative water contents (RWC) (d) in *Cinnamomum migao* seedlings treated with different inoculation treatments (NM, Cl, Ce, and HA) and different inoculum sizes (S1, S2, S3, and S4). *Note*: Vertical bars represent the standard errors of the means based on four replicates. Different lowercase letters indicate significant differences between different inoculum sizes in the same AMF treatment, whereas different capital letters indicate significant differences between different AMF treatments with the same inoculum size (LSD test; *p* < .05). Significant differences at **p < .001 and *p < .05, and NS indicates no significant differences

Under drought stress, TSS, SP, Pro, and RWC contents in *C. migao* seedlings increased to varying degrees depending on the differences in AMF and inoculum sizes. The contents of the four osmoregulatory substances first increased and then decreased with an increase in the inoculum size (Figure 5a–d).

TSS content in AM plants ranged from 0.3 ± 0.01% to 0.4 ± 0.01% (Figure 5a), which was significantly higher than that in NM plants by 8.3–90.5% (*p* < .05). Differences in AMF treatments had no significant effects on TSS content, whereas differences in inoculum sizes and the interaction between AMF and inoculum sizes significantly affected TSS content (*p* < .001; Figure 5a). SP content in AM plants ranged from 8.6 ± 0.2 to 13.5 ± 0.8 mg/g FW (Figure 5b). SP content in Cl‐S3 (12.1 ± 0.4 mg/g FW), Ce‐S3 (12.8 ± 0.3 mg/g FW), HA‐S2 (12.6 ± 0.3 mg/g FW), HA‐S3 (13.5 ± 0.8 mg/g FW), and HA‐S4 (12.5 ± 0.4 mg/g FW) plants was significantly higher than that in NM plants (*p* < .05; Figure 5b). Furthermore, SP content in plants receiving other treatments was also higher than that in NM plants, but the difference was not significant (Figure 5b). AMF and inoculum sizes had significant effects on SP content (*p* < .05), but the effect of their interaction was not notable in the two‐factor ANOVA (Figure 5b). Under drought stress, Pro content in AM plants ranged from 17.4 ± 1.7 to 35.4 ± 2.5 μg/g FW, which was significantly higher than that in NM plants by 96.8–335.6% (*p* < .05; Figure 5c). Significant differences were noted among the different inoculum sizes in the three AM plants (Cl, Ce, and HA) (*p* < .001; Figure 5c), demonstrating that Pro content was greatly affected by inoculum sizes. RWC in AM plants (65.5–91.4%) was higher than that in NM plants (62.4–71.3%) by 11.4–38.9% (*p* < .05; Figure 5d). Differences in AMF treatments had no significant effects on RWC, whereas differences in inoculum sizes and the interaction between AMF and inoculum sizes significantly affected SP content, as revealed by ANOVA (*p* < .05; Figure 5d).

### 3.7 Correlation between measurement indices

**FIGURE 6:**
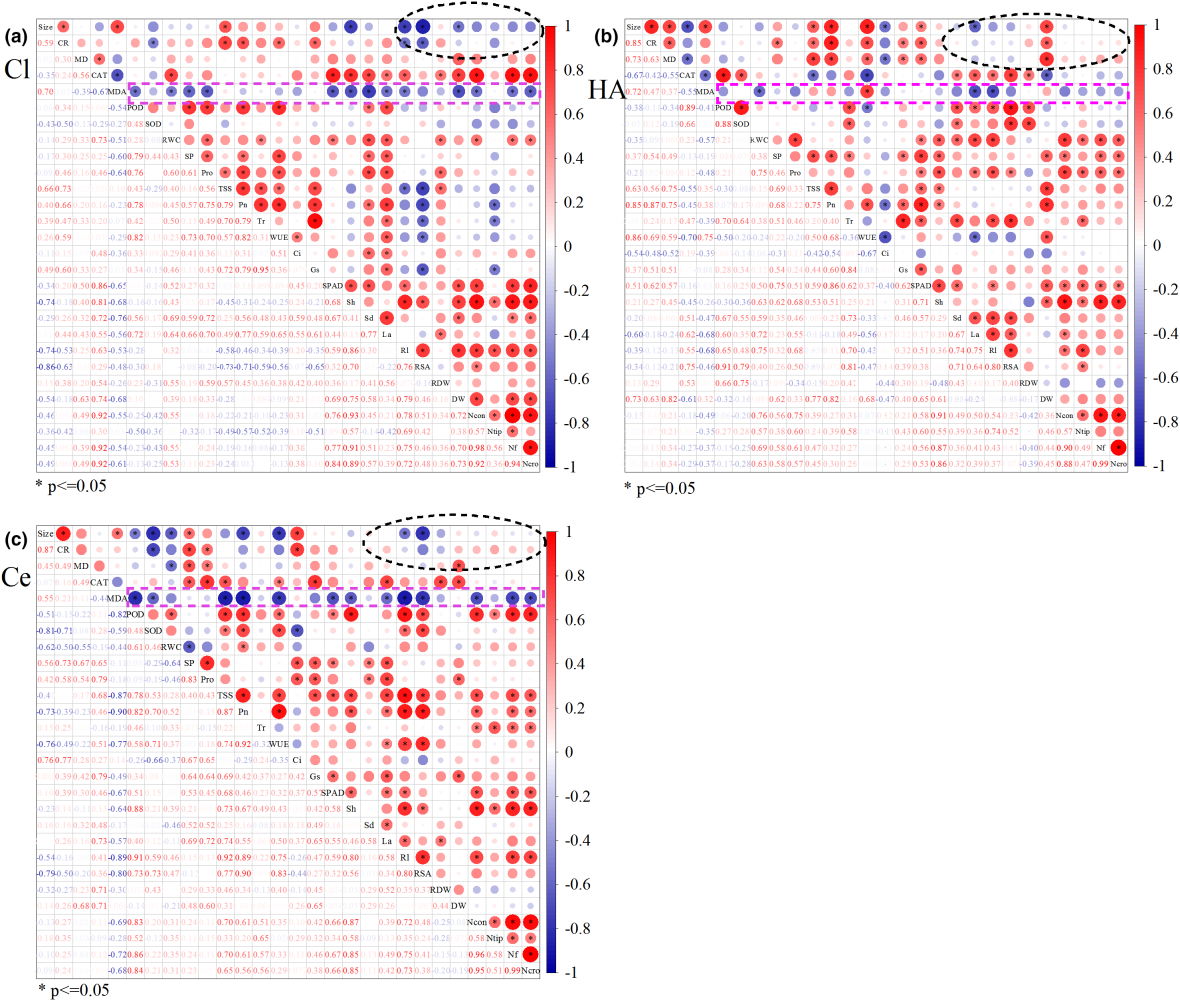
Pearson's correlation coefficients among inoculum sizes (size), *Cinnamomum migao* seedling mycorrhizal colonization rate (CR), growth characteristics, antioxidant enzyme activities, osmoregulatory substances, and photosynthetic parameters. (a) Groups inoculated with *Claroideoglomus lamellosum*; (b) groups coinoculated with *Claroideoglomus lamellosum* and *Claroideoglomus etunicatum*; and (c) groups inoculated with *Claroideoglomus etunicatum*

Correlation analysis revealed a significant positive correlation (*p* < .01) between inoculum size and mycorrhizal CR. The most indices of antioxidant enzymes, osmoregulatory substances, photosynthetic parameters, and plant growth also showed positive correlations; however, these indices were negatively correlated with MDA (Figure 6a–c). These results indicated that the symbiotic relationship between the plant and AMF could reduce the accumulation of MDA and prevent plant damage by broadly regulating antioxidant enzyme activity, photosynthetic parameters, and plant growth during drought stress. Notably, except for the DW of HA treatment, there was a mild negative correlation between inoculum size and most of biological growth indices in the three AM plants, which eventually became significantly negative as the inoculum size increased (*p* < .01, *p* < .05; Figure 6a–c). These results indicated that an excessive number of AMF inhibit plant growth, possibly because the nutrient competition overrules the nutrient synergy between the symbionts. Overall, the correlations between various indices of the three AMF treated plants were different, the indices of antioxidant enzymes, photosynthesis, and plant growth showed a stronger correlation in the HA plants (Figure 6b) and Ce plants (Figure 6c) than in the Cl plants (Figure 6a).

## 4 DISCUSSION

Arbuscular mycorrhizal fungi can form symbiotic relationships with most terrestrial plants and play an important role in plant growth and adaptation to various stresses (Brundrett & Tedersoo, 2018; Huang et al., 2020). Generally, mycorrhizal symbioses improve the growth of host plants due to direct H_2_O and nutrient absorption and transportation through mycorrhizal hyphae (Augé et al., 2015; Ruiz‐Lozano, 2003). The increase in inoculum sizes promoted mycorrhizal CR (Figure 2a) and enhanced MD (Figure 2b), which showed that the CRs of *Cl. etunicatum* and *Cl. lamellosum* in the roots of *C. migao* were high and that they easily formed a symbiotic relationship. In addition, previous studies reported that the symbiotic relationship formed by different AMF and the same host plant demonstrated differences in the physiological effects of the mycorrhiza (Zhang et al., 2011), which were caused by the biological characteristics of the species and the affinity between the species and host plant (Liu et al., 2006; Zhang et al., 2010), showing differences in MD. This study also confirmed the differences in the MD of plants treated with *Cl. lamellosum*, *Cl. etunicatum*, and their combination at the same inoculum size.

Furthermore, studies have shown that plants could enhance their access to various resources and occupy diverse environments by altering functional organ morphology, biomass allocation, and physiological characteristics (Ahmad et al., 2018; Brouwer, 1983). In this study, the inoculation of AMF facilitated root elongation (Table 2) and branching (Table 3), which enabled root growth in the soil and facilitated nutrient and water uptake. Numerous studies have also shown that modifications in the root architecture induced by AMF help maintain water status and essential nutrients (Aroca et al., 2013; Rajtor & Piotrowska‐Seget, 2016; Tamayo‐Velez & Osorio, 2017). Additionally, leaf characteristics and biomass were significantly improved (Table 2). Root and leaf structures are very important for the plant to resist drought stress (Ekblad & Högberg, 2001). Similar results were reported in previous studies – Boutasknit et al. (2020) and Sebastiana et al. (2018) found that with AMF inoculation, *Ceratonia siliqua* and cork oak root biomass and growth status were significantly improved in drought stress conditions.

The drought tolerance of a plant is determined by comprehensive physiological indices. Photosynthesis is the basis of plant productivity and crop yield and is highly sensitive to environmental factors (Yin & Struik, 2017). Under water‐deficit conditions as well as stomatal (reduced CO_2_ supply due to stomatal closure) and nonstomatal (decreased photosynthetic activity of mesophyll cells) factors lead to a decrease in the net Pn of plants (Deng et al., 2020; Farquhar & Sharkey, 1982). In this study, the inoculation of AMF improved Pn, WUE, Gs, SPAD, and Ci of *C. migao* seedlings, and decreased Tr (Figure 3a–f). Within a certain range of inoculum size (20–60 g), photosynthetic parameters (Figure 3a–f) corresponded to the change in mycorrhizal CR (Figure 2a), indicating that the colonization of AMF improved the drought tolerance of plants by affecting photosynthetic characteristics. Additionally, several studies on other AMF and plants have also confirmed similar results. They found that the colonization of AMF promoted plant growth and biomass accumulation under drought stress by improving photosynthesis (Caravaca et al., 2003; Mo et al., 2016; Smith & Smith, 2011).

This study found that the inoculation of *Cl. lamellosum* and *Cl. etunicatum* was beneficial in reducing MDA content (Figure 4d) and enhancing antioxidant enzyme activity (Figure 4a–c) in plants, indicating that AM plants developed mechanisms to reduce oxidative damage under drought stress. The establishment of an AM symbiosis and production of AM signals might have activated the antioxidant enzyme genes to counteract ROS‐mediated oxidative damage and maintain homeostasis in the plants under the conditions of water deficit (He et al., 2017; Huang et al., 2020; Jadrane et al., 2020; Ramli et al., 2020; Wang, Sun et al., 2019). Numerous studies have shown that oxidative stress occurs as a result of increased ROS and/or decreased capacity of the antioxidant system (Nawaz et al., 2017; Zhao et al., 2018). MDA content is an important index of plasma membrane damage (Loutfy et al., 2012). When plants were subjected to drought stress, the accumulation of ROS in the cell disrupted the metabolic balance, increased the MDA content and cell membrane permeability, damaged the cell structure, and affected the growth and development of plants (Krasensky & Jonak, 2012; Lopes et al., 2017). To survive under adverse environmental conditions, plants maintain an equilibrium between the formation and detoxification of ROS by activating the antioxidant defense system (comprising enzymatic and nonenzymatic antioxidants) to mitigate oxidative damage (Hossain et al., 2020). The antioxidant enzymes include CAT, POD, and SOD, which play important roles in mitigating oxidative damage (Halo et al., 2015; Parviz et al., 2015). SOD catalyzes the transformation of accumulated ROS into H_2_O_2_ and molecular oxygen species, whereas CAT and POD convert H_2_O_2_ into water and molecular oxygen for the elimination of ROS (Gao et al., 2018; Ragupathy et al., 2016; Sousa et al., 2020). In addition, the variation in SOD activity (Figure 4a) in AM plants was lower than that in POD (Figure 4c) and CAT (Figure 4b) activities at the inoculum sizes of 40 and 60 g, potentially because POD and CAT had a higher capacity for the decomposition of H_2_O_2_ generated by SOD (Cai & Gao, 2020) or because SOD, CAT, and POD had different sensitivities to different strains and inoculum sizes (Li et al., 2016). Hu et al. (2016) proposed that the maintenance of higher POD and CAT activities may provide further oxidative protection by detoxifying H_2_O_2_ produced due to stress by weakening the SOD enzyme system. Certainly, further research is warranted to reveal the mechanism by which AM symbiosis alters the adaptability of *C. migao* to drought tolerance, the relationship between AMF and the metabolic pathways of antioxidant enzymes, and the mechanism by which AM symbiosis regulates antioxidant enzyme gene expression.

During water shortage, plants altered the osmotic potential and increased the contents of osmoregulatory substances through osmotic regulation to sustain the balance between water content and cell swelling pressure, maintaining normal growth and metabolism to resist damage from drought (Zhang et al., 2017). The accumulation of activated osmotic molecular compounds and ions in plant cells may reduce osmotic potential, causing water to move into the cell, thereby increasing cell turgor to resist water deficit (Farooq et al., 2009). The main osmoregulatory substances, such as Pro, TSS, and SP, were involved in the regulation (Jadrane et al., 2020; Lin et al., 2019) and could be used as indices to evaluate drought tolerance in many plant breeding programs (Yu et al., 2017; Zhao et al., 2021). In this study, AM plants had higher Pro, TSS, and SP contents than NM plants under drought stress (Figure 5a–c). Hence, AM plants had higher RWC than NM plants under drought stress (Figure 5d). This was beneficial to maintain cell turgor and enhance the ability of plants to resist drought stress (Jadrane et al., 2020). Our results corroborated those of previous studies (Kasim et al., 2013; Ruiz‐Lozano & Azcón, 1995; Saravanakumar et al., 2011).

**FIGURE 7:**
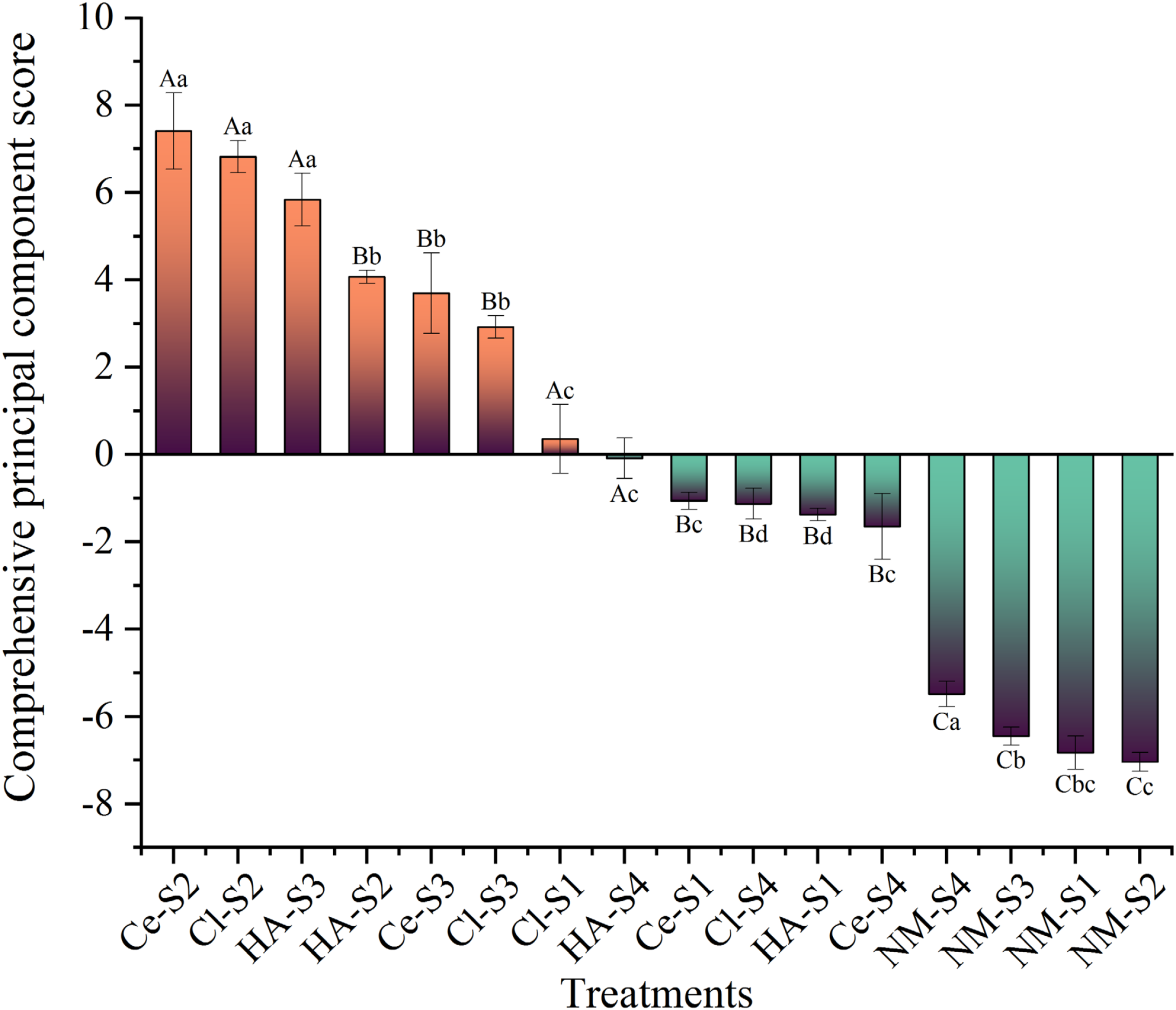
Ranking of comprehensive evaluation scores of drought tolerance of *Cinnamomum migao* seedlings with different treatments. *Note*: Vertical bars represent the standard errors of the means based on four replicates. Different lowercase letters indicate significant differences between different inoculum sizes in the same AMF treatment, whereas different capital letters indicate significant differences between different AMF treatments with the same inoculum size (LSD test; *p* < .05)

The effect of AMF colonization on host plants depends on many factors, including the host specificity and local diversity of AMF, the selectivity of host plants for AMF, and the adaptability of fungal strains to different environmental conditions, which affect inoculation effectiveness (Wei et al., 2020; Yang et al., 2012; Zhang et al., 2011). In this study, the inoculation of *Cl. lamellosum* and *Cl. etunicatum* increased the drought tolerance of *C. migao* seedlings. The three AMF inoculation treatments had significantly different effects on Sh, La, Rl, RSA, Ncon, Pn, WUE, Gs, SPAD, Ci, SOD, CAT, MDA, SP, and Pro, as revealed by ANOVA (Table 4). This indicated that *Cl. lamellosum*, *Cl. etunicatum*, and their combination improved the drought tolerance of *C. migao* seedlings via different mechanisms of action. The differences in the drought tolerance induced by AMF under the conditions of drought stress may be related to the biological characteristics of AMF and the origin of the strains (Smith & Read, 1997). Some studies showed that coinoculation produced better growth‐promoting effects than single inoculation (Takács et al., 2018) and that the inoculation of various types of fungi produced positive effects on target plants (Lioussanne et al., 2009). Similar results were obtained in the present study. At the 60 and 100 g inoculum size, coinoculation had a more positive effect on the drought tolerance of *C. migao* seedlings, but the effects were opposite at 20 and 40 g inoculum size. Meanwhile, the effects of the three AMF treatments at 40 and 60 g were significantly higher than those at 20 and 100 g (Figure 7). This might be caused by the interaction between the two fungi in antagonism of growth inhibition and competitive growth (Ansari & Ahmad, 2019). Coinoculation had a better inoculation effect as the inoculum size increased to 60 g, which might mean that the coexistence of the two microorganisms achieves a balance between survival and reproduction, and generates positive feedback to the plant symbiosis (Akiyama et al., 2002), thus showing a synergistic effect. However, when the inoculum size continued to increase, intraspecific competition as well as interspecific competition might exist between the two fungi, affecting substrate action (Morón‐Ríos et al., 2017; Zhang et al., 2010). The symbiosis between AMF and host plants was affected by intraspecific, interspecific, and nutrient competition between AMF and host plants (Johnson et al., 2012; Kiers et al., 2011; Lang et al., 2013; Morón‐Ríos et al., 2017), which was closely related to the quantity of AMF in the soil. Furthermore, inoculum sizes significantly affected CR, MD, plant growth indices, photosynthetic parameters, antioxidant enzymes, and osmoregulatory substances (Table 4), resulting in differences in drought tolerance. A low inoculum size (20 g) only partially induced growth, whereas appropriate inoculum sizes (40–60 g) had a better effect on plants and promoted drought tolerance (Figure 7). Conversely, a high inoculum size (100 g) inhibited plant growth. This was consistent with the results of Li et al. (2019). As the total fungal biomass increases, the net costs to the plant also increase because the mycorrhizal fungi can outcompete the plant for carbon to meet their energy demands (He et al., 2017; Johnson et al., 1997; Kiers et al., 2011). A symbiotic imbalance occurs when the net cost of symbiosis exceeds the net benefit (Liu et al., 2018), and in such situations, the mycorrhizal fungi may be considered parasitic to plants (Johnson et al., 1997). This nutrient competition affected the normal growth of plants (Huang, 2020). In addition, the interaction between AMF and inoculum sizes was obvious for all indices, excepting Gs and SP (Table 4). Therefore, it is necessary to investigate the appropriate concentration and inoculum size of different fungi types to promote the drought tolerance of *C. migao* seedlings.

**TABLE 4:** Two‐factor ANOVA results showing the effects of mycorrhiza (AMF), inoculum size, and their interactions on the listed variables

Treatments		AMF	Inoculum size	Interaction
				AMF × Inoculum size
df		2	3	6
Colonization rate (CR)	*F*	2.716	52.278	2.446
	*p*	NS	<.001	<.05
Mycorrhizal dependency (MD)	*F*	0.974	8.148	2.557
	*p*	NS	<.001	<.05
Shoot height (Sh)	*F*	39.073	56.373	52.833
	*p*	<.001	<.001	<.001
Stem diameter (Sd)	*F*	1.367	12.349	2.359
	*p*	NS	<.001	<.05
Leaf area (La)	*F*	5.303	33.363	3.86
	*p*	<.05	<.001	<.05
Root length (Rl)	*F*	36.015	103.685	13.82
	*p*	<.001	<.001	<.001
Root surface area (RSA)	*F*	44.148	61.694	12.007
	*p*	<.001	<.001	<.001
Root DW (RDW)	*F*	2.486	5.571	5.562
	*p*	NS	<.05	<.001
Total DW (DW)	*F*	2.117	8.935	9.609
	*p*	NS	<.001	<.001
Connection number of root (Ncon)	*F*	7.155	74.962	30.402
	*p*	<.05	<.001	<.001
Number of root tip (Ntip)	*F*	0.601	4.003	4.347
	*p*	NS	<.05	<.05
Number of root furcation (Nf)	*F*	2.804	53.674	32.184
	*p*	NS	<.001	<.001
Crossing number of root (Ncro)	*F*	1.68	37.166	24.541
	*p*	NS	<.001	<.001
Net photosynthetic rate (Pn)	*F*	35.337	111.914	97.342
	*p*	<.001	<.001	<.001
Water use efficiency (WUE)	*F*	5.85	6.037	22.956
	*p*	<.05	<.05	<.001
Stomatal conductance (Gs)	*F*	6.149	15.834	0.852
	*p*	<.05	<.001	NS
SPAD	*F*	20.562	68.947	17.274
	*p*	<.001	<.001	<.001
Transpiration rate (Tr)	*F*	0.475	13.919	5.341
	*p*	NS	<.001	<.001
Intercellular CO_2_ concentration (Ci)	*F*	59.685	11.264	28.916
	*p*	<.001	<.001	<.001
Superoxide dismutase (SOD)	*F*	33.676	26.345	36.578
	*p*	<.001	<.001	<.001
Catalase (CAT)	*F*	10.232	190.074	60.214
	*p*	<.001	<.001	<.001
Peroxidase content (POD)	*F*	2.435	33.286	13.652
	*p*	NS	<.001	<.001
Malondialdehyde (MDA)	*F*	5.042	54.836	2.911
	*p*	<.05	<.001	<.05
Total soluble sugars (TSS)	*F*	3.096	60.06	35.749
	*p*	NS	<.001	<.001
Soluble protein (SP)	*F*	4.218	5.033	0.86
	*p*	<.05	<.05	NS
Proline (Pro)	*F*	2112.843	3684.464	262.875
	*p*	<.001	<.001	<.001
Relative water content (RWC)	*F*	3.033	6.542	2.459
	*p*	NS	<.05	<.05

*Note*: NS, not significant at *p* = .05.

## 5 CONCLUSIONS

In this study, the inoculation of both *Cl. lamellosum* and *Cl. etunicatum* had positive effects on the drought tolerance of *C. migao* seedlings. Compared with NM plants, the roots of AM plants were able to establish symbiosis in association with the two AMF, thereby improving root growth. The well‐developed root network absorbed water and nutrients, maintaining water content and improving plant biomass, leaf photosynthesis, antioxidant enzyme activity, and osmoregulatory substance content, thereby mitigating ROS‐induced damage in plant cells. This study also showed that different inoculum sizes had significant effects on the drought tolerance of plants. As the inoculum size increased, host root CRs increased, whereas the most growth indices, photosynthetic parameters (Pn, WUE, Gs, SPAD, and Ci), antioxidant enzyme activities (SOD, CAT, and POD), and osmoregulatory substance contents (TSS, SP, Pro, and RWC) in *C. migao* seedlings first increased and then decreased. Conversely, MDA content first decreased and then increased. In summary, a low inoculum size (20 g) slightly improved plant growth, whereas medium inoculum sizes (40–60 g) had a greater effect on plant growth and promoted drought tolerance. Furthermore, a high inoculum size (100 g) may inhibit plant growth. When the inoculum size was ≥60 g, coinoculation had a more positive effect on the drought tolerance of *C. migao* seedlings than single inoculation, but the effect was opposite when the inoculum size was ≤40 g. Thus, it is of great significance to study the correlation between the inoculation density of AMF and drought tolerance of plants, which should be explored further in future studies. The results of this study revealed the mechanism by which AMF promote the drought tolerance of *C. migao* seedlings and provided a basis for further understanding the physiological and biochemical mechanisms by which different AMF and inoculum sizes result in the reduction of the damage caused to *C. migao* seedlings under drought stress. However, further studies are required to evaluate the role of *Cl. etunicatum* and *Cl. lamellosum* in improving plant growth and increasing resistance to drought stress under field and natural conditions.

## AUTHOR CONTRIBUTIONS

**Qiuxiao Yan:** Conceptualization (lead); data curation (lead); formal analysis (lead); methodology (lead); project administration (lead); visualization (lead); writing – original draft (lead); writing – review and editing (lead). **Xiangying Li:** Writing – original draft (supporting); writing – review and editing (equal). **Xuefeng Xiao:** Data curation (supporting); formal analysis (supporting); investigation (supporting); methodology (supporting); writing – review and editing (supporting). **Jingzhong Chen:** Formal analysis (supporting); investigation (supporting); methodology (supporting). **Jiming Liu:** Conceptualization (equal); funding acquisition (lead); project administration (supporting); supervision (supporting); validation (supporting); writing – review and editing (supporting). **Changhu Lin:** Conceptualization (supporting); supervision (supporting); writing – review and editing (supporting). **Ruiting Guan:** Formal analysis (supporting); investigation (supporting); methodology (supporting). **Daoping Wang:** Data curation (supporting); writing – original draft (supporting); writing – review and editing (supporting).

## CONFLICT OF INTEREST

The authors declare that they have no conflicts of interest.

## Data Availability

Data used in this study are stored in Dryad (https://doi.org/10.5061/dryad.5dv41ns88).
